# Virtual Reality as a Non-Pharmacological Aid for Reducing Anxiety in Pediatric Dental Procedures

**DOI:** 10.3390/children12070930

**Published:** 2025-07-14

**Authors:** Laria-Maria Trusculescu, Dana Emanuela Pitic, Andreea Sălcudean, Ramona Amina Popovici, Norina Forna, Silviu Constantin Badoiu, Alexandra Enache, Sorina Enasoni, Andreea Kiș, Raluca Mioara Cosoroabă, Cristina Ioana Talpos-Niculescu, Corneliu Constantin Zeicu, Maria-Melania Cozma, Liana Todor

**Affiliations:** 1 Faculty of Dental Medicine, Victor Babes University of Medicine and Pharmacy of Timisoara, Eftimie Murgu Sq., 300041 Timisoara, Romania; laria.trusculescu@umft.ro (L.-M.T.); dana.emanuela@gmail.com (D.E.P.); cosoroaba.raluca@umft.ro (R.M.C.); ioana.talpos-niculescu@umft.ro (C.I.T.-N.); 2 Department of Ethics and Social Sciences, George Emil Palade University of Medicine, Pharmacy, Science and Technology of Targu Mures, 540142 Târgu Mureș, Romania; melaniacozma76@gmail.com; 3 Department of Implantology and Prosthetic Implant Rehabilitation, Faculty of Dental Medicine, “Grigore T. Popa” University of Medicine and Pharmacy, 700946 Iasi, Romania; norina.forna@umfiasi.ro; 4 Department of Anatomy and Embryology, Faculty of General Medicine, UMFCD, Dionisie Lupu Str., nr.37, Sector 2, 020021 Bucharest, Romania; silviu.badoiu@umfcd.ro; 5 Discipline of Legal Medicine, Bioethics, Deontology and Medical Law, Faculty of Medicine, Victor Babeș University of Medicine and Pharmacy of Timisoara, Eftimie Murgu Sq., 300041 Timișoara, Romania; enache.alexandra@umft.ro; 6 Faculty of Medicine, Doctoral School, Victor Babeș University of Medicine and Pharmacy of Timisoara, Eftimie Murgu Sq., 300041 Timisoara, Romania; sorina.enasoni@umft.ro; 7 Research Center for Pharmaco-Toxicological Evaluations, Faculty of Pharmacy, “Victor Babes” University of Medicine and Pharmacy, Eftimie Murgu Sq., No. 2, 300041 Timisoara, Romania; kis.andreea@umft.ro; 8 Private Medical Office, 325400 Caransebes, Romania; drzeicucorneliu@gmail.com; 9 Departament of Dental Medicine, Faculty of Medicine and Pharmacy, University of Oradea, 10 Decembrie Sq., 410068 Oradea, Romania; liana.todor@gmail.com

**Keywords:** virtual reality, non-pharmacological anxiety reduction, dental anxiety, pediatric dentistry

## Abstract

Background/Objectives: Dental anxiety in children is a common issue that can hinder the delivery of effective dental care. Traditional approaches to managing this are often insufficient or involve pharmacological interventions. This study shows the potential of virtual reality (VR) to aid in reducing anxiety in children undergoing simple dental procedures. By immersing children in relaxing VR environments (such as beaches, forests, mountains, or underwater scenes with calm music), the objective is to assess VR’s effectiveness in calming pediatrics patients during these procedures. Methods: Children scheduled for minor dental treatments wore a wearable device that monitored pulse, perspiration, and stress levels. Each child’s baseline data was collected without the VR headset, followed by data collection during VR exposure before and during dental procedures. VR scenarios ranged from soothing nature scenes to animated cartoons, designed to foster relaxation. Results: The data collected showed a reduction in physiological indicators of stress, such as lower heart rate and reduced perspiration, when the VR headset was used. Children appeared more relaxed, with a calmer response during the procedure itself, compared to baseline levels without VR. Conclusions: This study provides preliminary evidence supporting VR as an effective tool for reducing anxiety and stress in pediatric dental patients. By offering an engaging, immersive experience, VR can serve as an alternative or complementary approach to traditional anxiety management strategies in pediatric dentistry, potentially improving patient comfort and cooperation during dental procedures. Further research could determine if VR may serve as an alternative to local anesthesia for non-intrusive pediatric dental procedures.

## 1. Introduction

Anxiety, as an emotional response, is a person’s specific way of responding to stress, which often persists after a perceived traumatic event has passed.

Dental anxiety in children is defined as an excessive or unreasonable fear associated with dental visits or procedures, often resulting in avoidance behaviors, distress, or poor cooperation during treatment. It is frequently rooted in prior negative experiences, fear of pain, or unfamiliarity with the clinical environment [1].

Anxiety is a prevalent challenge in pediatric dentistry, as in many other medical specialties that involve physical intervention, affecting up to 20% of children and significantly impacting their cooperation and the quality of care provided [2]. On the other hand, this disorder has a significant impact on the quality of life of patients, or can generate in the short or long term other comorbidities such as anxiety disorders, including medical phobias.

Special or unusual life contexts or events, such as the COVID-19 pandemic, interfere with acceptance and adherence to treatments [3,4,5]. Patients, especially those suffering from other medical conditions such as diabetes, cancer, autoimmune, or inflammatory diseases, could experience worsened anxiety symptoms and phobias towards medical procedures, resulting in medical complications, including dental ones [6,7,8,9].

Traditional methods of managing this anxiety, such as the “tell-show-do” technique, audio-visual distractions, or pharmacological interventions, often yield inconsistent results or introduce risks associated with medication, such as side effects and potential suicidal thoughts [10,11,12,13,14]. These limitations underscore the need for innovative non-pharmacological approaches to alleviate anxiety and improve the dental experience for children.

Virtual reality (VR) refers to the use of computer-generated environments that simulate real or imagined scenarios, allowing users to experience immersive sensory input through visual, auditory, and sometimes haptic feedback. In medical settings, VR is increasingly applied as a distraction and therapeutic tool, particularly for anxiety and pain management [15].

Virtual reality has emerged as a promising solution, leveraging immersive technology to distract and calm patients during dental procedures [16,17]. This method, like other innovative ways that emphasize personalization of medical care, must take into account certain indicators regarding individual characteristics, beliefs and convictions, desires and affects, and so on [18].

These investigations highlight VR’s ability to immerse young patients in calming environments, effectively distracting them from the anxiety-inducing aspects of dental care. To address this gap, the present study explores the use of VR to manage anxiety during minor dental procedures in children. This research builds on prior findings by integrating biometric monitoring to measure stress responses, including heart rate and skin-conductance, providing a robust dataset for evaluating VR’s efficacy.

Unlike conventional audio-visual tools, VR offers a multisensory environment that can fully engage the patient’s attention, effectively diverting focus away from the clinical setting. Several studies have shown that VR can reduce physiological indicators of stress, such as heart rate and cortisol levels, while fostering a more positive attitude toward medical care [15,19,20]. This capability positions VR as a transformative tool in pediatric dentistry, providing both behavioral and emotional relief during potentially anxiety-inducing procedures.

Constantini Leopardi et al. argue that managing anxiety in pediatric dentistry is crucial for achieving favorable treatment outcomes [21,22]. They contend that traditional methods, such as oral analgesics, fail to address anxiety effectively, leading to poor cooperation and delays in care. They introduce virtual reality (VR) goggles as an emerging distraction technique, citing studies that demonstrate its ability to reduce anxiety by isolating children from visual and auditory stimuli associated with dental procedures. They further emphasize that the multisensory distraction provided by VR goggles enhances its superiority over traditional techniques. Unlike passive approaches like music or TV, VR creates an immersive environment, redirecting the child’s focus entirely. The authors highlight studies where VR significantly outperformed other distraction methods in reducing pain perception and anxiety during injections and surgical procedures. The same importance belongs to doctor–patient communication, an essential ingredient in the acceptance of the method and individual adaptation [23].

The authors of “An Interactive Augmented and Virtual Reality System for Managing Dental Anxiety among Young Patients: A Pilot Study” argue that pediatric dental anxiety is a pervasive issue that hinders effective treatment and increases stress for patients, parents, and dentists [24]. They assert that traditional methods like the tell-show-do (TSD) technique and audio-visual distractions are insufficient to address this widespread problem. They propose using an augmented and virtual reality system called “Dr. Barea” to familiarize children with dental environments and reduce anxiety through immersive and interactive experiences. The authors suggest that VR’s ability to simulate a 360-degree dental clinic environment and AR’s capacity to educate children about dental tools are its key advantages.

Critics might claim that existing distraction methods are adequate for managing dental anxiety, but the authors counter this by emphasizing the lack of engagement in traditional techniques. They cite studies demonstrating VR’s and AR’s effectiveness in fostering familiarity with dental procedures, which in turn reduces fear. For instance, they presented a feasibility study involving 16 children, where those exposed to the VR system showed a statistically significant decrease in anxiety levels compared to the control group. Recent studies also indicate that VR systems can be beneficial in reducing anxiety levels among neurodivergent children [25,26].

The authors, Rosa et al., argue that dental phobia in children is a significant barrier to achieving optimal oral health and dental care [27]. They assert that traditional methods, such as pharmacological and behavioral techniques, fall short in managing anxiety effectively. Introducing virtual reality (VR) as a therapeutic tool, they claim, provides an immersive and interactive environment capable of reducing anxiety and pain by distracting children during dental treatments. The authors base their arguments on a systematic review of 11 studies involving children under 18 years old.

Critics might suggest that VR is just one of many distraction methods and may not outperform simpler, less costly techniques like music or audio-visual aids. However, Rosa et al. counter this by highlighting VR’s unique advantages, including its ability to fully immerse patients in a calming, three-dimensional environment. They reference studies that document VR’s superiority in lowering self-reported anxiety scores and physiological stress markers compared to traditional methods [27]. The authors emphasize that these immersive experiences help children feel in control, which is key to reducing dental anxiety.

They also address challenges to VR’s implementation, such as its cost and accessibility. Some researchers argue that these factors limit VR’s feasibility in widespread clinical use. The authors acknowledge these criticisms but advocate for the integration of VR with existing behavioral management techniques to maximize its impact. They highlight studies showing that VR’s benefits are particularly pronounced in pediatric populations as children are more engaged and responsive to immersive technologies than adults [28].

The integration of virtual reality (VR) into pediatric dentistry represents a significant step forward in non-pharmacological approaches to managing dental anxiety [29]. By immersing children in calming, engaging environments, VR offers a novel way to divert their attention from stress-inducing stimuli, fostering a sense of safety and relaxation. The findings from previous studies consistently demonstrate the effectiveness of VR in reducing physiological indicators of stress, such as heart rate and cortisol levels, and improving behavioral responses during dental procedures. These advantages not only enhance patient comfort but also facilitate smoother, more effective treatments.

Nonetheless, the evidence suggests that VR has the potential to transform pediatric dental care by offering an effective, child-friendly alternative to traditional anxiety management techniques. As research continues to expand, VR may also serve as a complementary tool to existing methods or even reduce reliance on pharmacological interventions for non-invasive procedures. By bridging the gap between patient comfort and clinical efficacy, VR holds the potential to redefine the pediatric dental experience, paving the way for more accessible, anxiety-free dental care for children.

The purpose of this study is to evaluate the effectiveness of virtual reality in reducing dental anxiety and physiological stress among children aged 6–12 undergoing minor dental procedures. Given the promising potential of virtual reality (VR) in managing pediatric dental anxiety, it becomes imperative to explore its practical applications and assess its efficacy through structured and measurable approaches. This study builds on the existing body of evidence by implementing a controlled experimental design to evaluate the physiological and behavioral effects of VR during dental procedures in children. By utilizing wearable devices to monitor stress indicators and carefully designing immersive VR scenarios, the research seeks to provide quantitative insights into the effectiveness of this technology. The following section outlines the materials and methods employed to conduct this study, detailing the tools, protocols, and parameters used to ensure accurate and reliable results.

The interest in non-pharmacological methods for managing anxiety in pediatric settings is an important aspect of medical care. In this light, it is important to investigate the mechanisms through which virtual reality (VR) may exert its anxiolytic effects. Several mechanisms may underlie the effect. First, immersive distraction diverts attentional resources away from nociceptive or anxiety-provoking cues, in line with the gate-control framework [30]. Second, VR provides rich sensory inputs and agency, which can foster a sense of presence that overrides situational stressors. Finally, engaging, game-like tasks may elicit positive effects and thereby down-regulate arousal via pre-frontal inhibitory pathways. In this context, the present study aims to evaluate the impact of VR-assisted procedures on anxiety levels in neurodivergent children undergoing dental visits, comparing their responses across sessions with and without VR exposure.

## 2. Materials and Methods

This study introduces a diverse range of VR content tailored to the preferences and needs of pediatric patients, offering a comprehensive approach to enhancing the dental experience.

The study’s methodology was designed with replicability in mind, incorporating consistent protocols across multiple clinical settings to ensure generalizable results. By comparing biometric and behavioral outcomes in sessions with and without VR, this research aims to quantify VR’s impact and identify its potential as an alternative or complementary tool to traditional anxiety management techniques.

In the following section, the materials and methods used in this study are described in detail. The section outlines participant recruitment, experimental design, the biometric tools employed, and the implementation of VR during dental procedures. This structured approach ensures clarity and reproducibility for researchers seeking to build on these findings in future studies.

This study employed a controlled experimental design to evaluate the effectiveness of virtual reality (VR) as a non-pharmacological tool for reducing anxiety in pediatric dental procedures. Conducted between September and November 2024, the study involved 120 children aged 6–12 years, recruited from three dental clinics located in Timișoara, Romania. Each child participated in two sessions, one without VR and another with VR, separated by a six-week interval to minimize carryover effects. Written informed consent was obtained from all participants’ parents or guardians, including consent for data collection and publication. Additionally, GDPR-compliant agreements ensured the protection and confidentiality of the collected data.

Participants were randomly selected based on the following inclusion criteria:

- Aged between 6 and 12 years;
- Scheduled for non-invasive dental procedures, such as dental cleanings, fluoride applications, or examinations for early-stage caries;
- No history of severe dental phobia, epilepsy, vestibular or cognitive disorders, or other neurological conditions that may contraindicate VR use;
- Children with visual or auditory impairments, diagnosed anxiety disorders, or those requiring extensive dental treatments were excluded from the study.

### 2.1. Data Collection

Each participant underwent two dental sessions: one without VR intervention (control) and one with VR (intervention). Both sessions followed identical protocols, ensuring consistency in dental procedures and monitoring conditions. Sessions were conducted on different days to minimize carryover effects from prior experiences.

To measure stress and physiological responses participants wore a biometric bracelet throughout each session (E4 wristband model). The bracelet recorded the following:

o EDA (electrodermal activity): Refers to changes in the electrical properties of the skin, primarily due to sweat gland activity. It is measured in microsiemens (μS) and reflects the level of emotional arousal or stress. Higher EDA values typically indicate increased sympathetic nervous system activation.
o BVP (blood volume pulse): Measures changes in blood volume in peripheral circulation, usually obtained through photoplethysmography. It is often represented in arbitrary units and provides information about heart rate and blood flow. BVP patterns can indicate cardiovascular responses to stress or relaxation.
o Stress level: This is not directly measured but inferred from physiological indicators like EDA and BVP. Increased stress is generally associated with higher EDA values, more frequent EDA peaks, and greater variability in BVP patterns. Stress levels are typically described qualitatively (low, medium, high) based on combined analysis.

Data were collected in real-time and transmitted securely to a dedicated software system for analysis. Baseline biometric readings were recorded for five minutes before the procedure in a relaxed seated position.

During the VR intervention session, participants wore a lightweight, child-friendly VR headset (Kit Limbix VR model). The headset displayed calming content, including the following:

1. Animated cartoons such as SpongeBob SquarePants, Tom and Jerry, etc.;
2. Immersive natural scenes, such as beaches, forests, or underwater environments, accompanied by soothing instrumental music.

Content was pre-selected to ensure age-appropriateness and avoid overstimulation or discomfort. Participants were allowed to choose between available options, enhancing engagement and comfort.

All participants underwent small, non-invasive dental procedures lasting 10–15 min. Examples of these procedures include the following:

1. Routine dental examinations;
2. Fluoride application;
3. Professional dental cleanings (supragingival scaling).

Procedures were performed by experienced pediatric dentists using standardized techniques to maintain uniformity across the three clinics.

Biometric data were collected continuously during the baseline period, the dental procedure, and a five-minute post-procedure recovery phase. For the control session, participants did not wear the VR headset but were encouraged to remain calm through verbal reassurance. In the VR session, biometric data were recorded under identical conditions while participants viewed VR content.

Also important to mention is that to ensure safety all VR equipment underwent sterilization before use, and children were monitored for signs of cybersickness, such as dizziness or nausea. Post-procedure, children and parents provided qualitative feedback on their experience, including comfort levels and perceived anxiety during the session.

### 2.2. Study Objectives and Purpose

The primary objective of this study is to evaluate the efficacy of virtual reality (VR) as a non-pharmacological tool for reducing anxiety and stress in pediatric patients undergoing minor dental procedures. Specifically, the study aims to do the following:

1. Quantify physiological effects: Assess the impact of VR on physiological stress indicators, such as heart rate, heart rate variability, and skin-conductance, compared to a control condition without VR. Moreover, to determine whether the use of VR during dental procedures improves the comfort, relaxation, and cooperation of pediatric patients.
2. Compare VR to traditional techniques: Contrast the outcomes of VR interventions with traditional approaches for managing pediatric dental anxiety to establish its potential as an alternative or complementary method.
3. Support future applications: Provide preliminary evidence to inform the broader adoption of VR in clinical pediatric dentistry and guide future research on its long-term impacts and applicability for more invasive procedures.

Strengths of the study include a controlled experimental design with pre- and post-procedure biometric monitoring; the use of age-appropriate and customizable VR content to enhance engagement and comfort; and the integration of qualitative feedback from both children and their parents to gain insights into perceived anxiety levels. By focusing on non-invasive dental procedures and tailoring the VR experience to the psychological needs of children aged 6–12, the study ensures developmental appropriateness.

### 2.3. Limitations and Potential Bias

1. Sample size and diversity: The study included 120 participants from three clinics, which may not represent broader demographic or geographic variability. Results might differ in other populations or cultural contexts.
2. Non-blinded design: Participants and dental practitioners were aware of the intervention (VR use), which could introduce performance or placebo effects.
3. Short-term evaluation: The study focused on immediate physiological and behavioral responses during and shortly after the dental procedure. Long-term effects on dental anxiety reduction were not assessed. Moreover, each child participated in only one session with VR and one without, which may not capture the cumulative effects of repeated VR use over time.
4. Variability in VR content preferences: Individual differences in preferences for VR content (cartoons vs. natural scenes) could influence engagement levels and outcomes, introducing variability in the results.
5. Limited range of dental procedures: The study focused on minor, non-invasive dental procedures, which may not fully represent the anxiety levels experienced during more complex or painful treatments.
6. Physiological measurement constraints: Biometric data were collected via wearable devices that, while validated, might have limited accuracy compared to clinical-grade monitoring equipment.
7. Potential external influences: Factors such as parental presence, clinic environment, and interactions with dental staff could influence anxiety levels independently of VR use.
8. Standardization challenges across clinics: Although efforts were made to maintain consistency, differences in clinic environments, practitioner approaches, or equipment could have introduced variability.

## 3. Results

This section presents the results of our analysis, comparing the first dental session (conducted without VR intervention during participants’ initial voluntary visit) with the second session, which incorporated VR during their subsequent voluntary visit. The analysis reflects data collected from 120 participants. The data reveal distinct patterns in stress responses, providing insights into the potential benefits of VR in medical settings for children.

The information from the graphical outputs of the E4 bracelet has been synthesized into concise tabular formats. These tables provide a more accessible representation of the key physiological parameters measured during the sessions. By distilling the complex waveforms and time-series data into quantitative summaries, we aim to enhance the clarity and comparability of the electrodermal activity (EDA) and blood volume pulse (BVP) measurements across different experimental conditions. This approach allows for a more straightforward assessment of the physiological responses observed during the dental procedures, with and without virtual reality interventions.

### 3.1. Sample-Size Determination

A priori power analysis was conducted with G*Power 3.1.9.7 [31] in order to estimate the minimum number of participants required for the study. The analysis was performed using a *t*-test on the difference between two dependent means (matched pairs), and it was two-tailed because each child served as his or her own control (baseline vs. VR session).

Input parameters were chosen as follows:

- Effect size (d) = 0.30.

This value represents a small-to-medium change that was considered clinically meaningful on the basis of previous pediatric VR studies reporting within-subject effects in the range 0.25–0.40.

- α error probability = 0.05 (two-sided).
- Statistical power (1 − β) = 0.90, ensuring a 10% maximum risk of Type II error.

**Figure 1:**
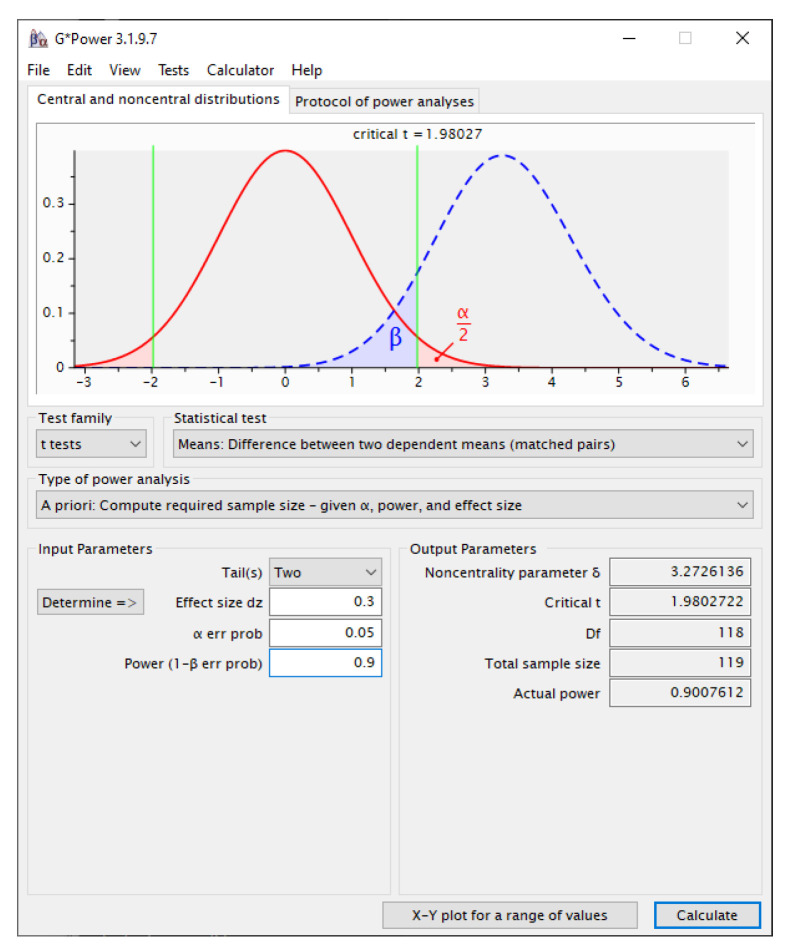
Screenshot from G*Power—sample calculation = 119.

With these specifications G*Power (version 3.1.9.7) returned a non-centrality parameter δ = 3.27, a critical t of 1.98, and a required total sample size of 119 paired observations (df = 118) to detect the target effect (Figure 1).

Because 120 children completed both measurement sessions, the achieved sample satisfied and slightly exceeded the a priori requirement, yielding an actual power of 0.91. No additional inflation for attrition was necessary as no data losses occurred after enrolment.

The a priori power analysis was based on an effect size of _d_z = 0.30.

This value reflects the lower bound of a clinically meaningful change in pediatric physiological and pain outcomes; systematic MCID reviews indicate that children reliably notice improvements of ~0.3 SD [32], and recent empirical work confirms that a 0.28–0.35 SD shift in vital-sign-linked pain measures is interpreted by pediatric patients as ‘adequate analgesia’ [33]. Accordingly, detecting a Δ of 0.30 ensures the study is powered to capture the smallest effect size that would justify implementation in routine clinical practice.

### 3.2. Statistical Analysis

All data were processed with IBM SPSS Statistics 25 [34] by a researcher who was not involved in data collection. The analytic workflow followed APA reporting recommendations and comprised the following two stages: descriptive and inferential statistics. [35]

#### 3.2.1. Descriptive Statistics

For each study condition—baseline without VR and intervention with VR—we obtained measures of central tendency and dispersion (M, SD, SE, and 95% CI). Distributions were illustrated with histograms and boxplots. The physiological outcomes were as follows:

- Electrodermal activity (EDA): Integer counts of skin-conductance responses per session (SCR peaks).
- Blood volume pulse (BVP): Integrated plethysmographic amplitude expressed in device-specific, arbitrary units (30–500).

No coding errors or extreme outliers (±3 × IQR) were detected; complete paired data were available for 120 participants.

#### 3.2.2. Inferential Statistics

- Normality check. The distribution of change scores was examined with the Shapiro–Wilk test. Given the large sample (n = 120), the paired-samples *t*-test was retained even when minor deviations from normality were present.
- Paired comparisons. Two-tailed paired-samples *t*-tests compared baseline and VR values for EDA and BVP. Statistical significance was set at α = 0.05. The output reported *t* values, degrees of freedom (df = n − 1), exact *p* values, and 95% CIs for the mean differences.
- Effect size. Practical importance was quantified with Cohen’s d for dependent means, calculated as d = t/√ n. Benchmarks of 0.20, 0.50, and 0.80 were interpreted as small, medium, and large effects, respectively.

All tests were two-sided and no adjustment for multiplicity was required because only two primary endpoints were analyzed. Results were considered statistically significant at *p* < 0.05 and are reported to two decimals for descriptive metrics and three decimals for inferential probabilities (or “<0.001” when appropriate), in accordance with APA 7th edition guidelines [35].

### 3.3. Statistical Results

Pre-analysis screening: A visual inspection of boxplots revealed no extreme outliers. All 120 children provided complete paired data for both outcome variables.

Assumption checking: Normality of the change scores (VR − No-VR) was assessed with the Shapiro–Wilk test.

**Figure 2:**
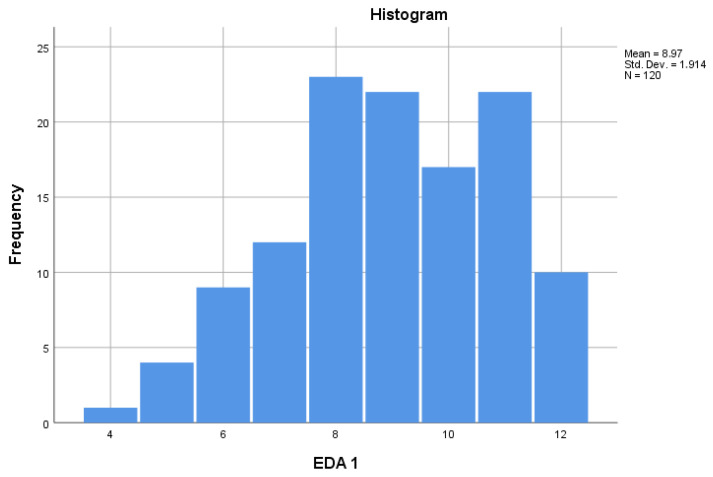

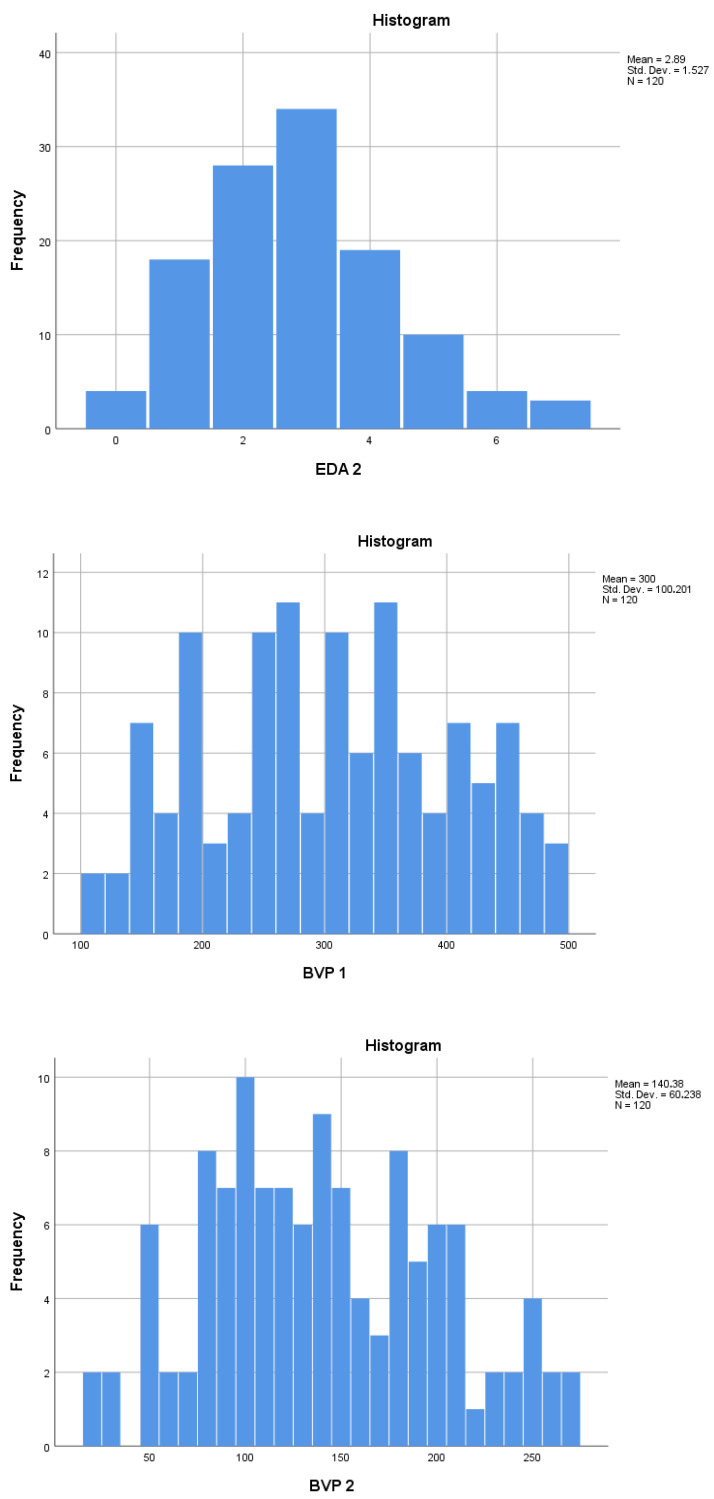
Distribution and normality of change scores.

EDA differences deviated from normality, W = 0.88 and *p* < 0.001, whereas BVP differences were approximately normal, W = 0.98 and *p* = 0.059. Given the large sample size, the paired-samples *t*-test was judged sufficiently robust to these departures (Figure 2).

#### 3.3.1. Descriptive Statistics

Mean (M) and standard deviation (SD) for each condition are displayed in Table 1. On average, children exhibited fewer electrodermal responses and lower blood volume pulse amplitudes during the VR session than during the baseline.

#### 3.3.2. Paired Comparisons

Two-tailed paired-samples *t*-tests confirmed that both physiological indices decreased significantly in the VR condition (Table 2).

Cohen’s d was derived from the t statistic (d = t/√n), yielding very large effects for both EDA (d = 3.84) and BVP (d = 2.77).

#### 3.3.3. Summary

Electrodermal reactivity dropped by approximately 68% and blood volume pulse amplitude by about 53% when children were exposed to the VR distraction. The effects were statistically significant (*p* < 0.001) and exceeded conventional benchmarks for a large practical effect, indicating a robust physiological calming impact of the VR intervention.

Physiologically, fewer EDA peaks reflect diminished sympathetic sudomotor activity, whereas a lower BVP amplitude suggests reduced peripheral vasoconstriction and cardiac output. Together, the data point to a robust attenuation of sympathetic nervous-system activation during the VR sessions. These findings align with the broader VR literature documenting reductions in pain, anxiety, and distress in pediatric medical contexts [35,36], but extend prior work by demonstrating objective autonomic changes rather than relying solely on self-reports.

## 4. Discussion

Children aged 6 to 12 years are in a critical stage of cognitive and emotional development, characterized by increasing independence, heightened emotional sensitivity, and evolving coping mechanisms [37]. At this age, children begin to understand cause–effect relationships but may still struggle with abstract reasoning and self-regulation under stress. Their psycho–emotional responses during dental treatments often reflect a mix of fear of the unknown, sensitivity to perceived pain, and strong reactions to environmental stimuli such as sounds, smells, or instruments. Behavioral responses may include crying, refusal to cooperate, muscle tension, or psychosomatic symptoms like stomach aches. Therefore, managing dental anxiety in this group requires both emotional reassurance and tools that align with their developmental stage, such as engaging distractions or familiarization techniques, which makes virtual reality particularly suitable [37].

The present study’s findings on the efficacy of virtual reality (VR) as a non-pharmacological aid for reducing anxiety during pediatric dental procedures align with and extend the growing body of literature on VR applications in pediatric healthcare [38,39]. Our results, demonstrating significant reductions in physiological stress indicators during VR-assisted dental procedures, corroborate the broader trends observed in the previously mentioned systematic reviews and meta-analyses.

There were significant reductions in electrodermal activity (EDA) and improved blood volume pulse (BVP) stability during VR-assisted sessions, confirming in this way the conclusions of other studies in similar fields [36].

The large effect sizes for anxiety reduction reported in our study are consistent with the findings of Romero-Ayuso et al. in their comprehensive meta-analysis of VR use across various pediatric medical procedures [36]. Their study found large effect sizes for measures of sustained attention and moderate effects for processing speed, suggesting that VR interventions can effectively target multiple cognitive domains across different pediatric populations. This broader context supports the generalizability of VR’s stress-reducing effects across different medical contexts, including dentistry.

Our study’s focus on objective physiological measures (EDA and BVP) addresses a critical gap in the existing literature, which has predominantly relied on subjective self-reports or observer ratings. This approach offers a more nuanced understanding of the immediate physiological impacts of VR interventions, complementing the subjective measures commonly used in previous studies. This aligns with the work of Tsolakis et al., who emphasized the importance of objective measurements in evaluating the effectiveness of advanced technologies in pediatric interventions [40].

The effectiveness of VR interventions in improving cognitive performance without pharmacological intervention is a crucial finding. Given that there is no specific pharmacological therapy for dental anxiety in children, our results underscore the potential of VR as a non-pharmacological intervention that can significantly improve patient comfort and cooperation during dental procedures. This is particularly relevant in light of the findings by Valls-Esteve et al., who highlighted the potential of immersive technologies to provide more tailored and effective interventions for pediatric populations [41].

These findings have important implications for clinical practice in pediatric dentistry. The use of VR could offer a novel approach to complement traditional anxiety management techniques, potentially leading to more comprehensive and effective treatment strategies. Moreover, the ability of VR to provide personalized and adaptive interventions could address the diverse needs of pediatric patients, as suggested by Maresca et al. in their study on VR applications for children with dyslexia [42].

In the broader context of pediatric healthcare, our findings contribute to the growing evidence supporting the integration of VR technologies into clinical practice. As healthcare systems increasingly recognize the importance of patient experience and holistic care approaches, VR presents a promising, non-pharmacological tool for enhancing pediatric patient comfort and cooperation.

Future research directions should include exploring the long-term effects of VR use in pediatric dentistry, such as reducing dental phobia and improving overall oral health outcomes. Additionally, investigating the optimal design of VR content specifically for dental settings and different age groups could further enhance the effectiveness of these interventions, as suggested by the work on personalized VR interventions in other pediatric contexts [40,41,42].

While most pediatric VR studies report d values in the 0.3–1.0 range for subjective pain or anxiety, the current effects are substantially larger. The following two design features may account for this discrepancy: (a) objective physiology may capture subtle changes that children are unable to verbalize, and (b) the VR scenario used here was specifically tailored to the dental context, increasing ecological relevance.

Our study provides promising evidence for the effectiveness of VR-based interventions in reducing anxiety during pediatric dental procedures. These findings open new avenues for research and clinical applications in the field of pediatric dentistry, potentially improving the overall experience and outcomes for young patients.

## 5. Conclusions

From a clinical vantage, a single VR session produced pronounced autonomic quieting without adverse events or additional staff time. Head-mounted displays are becoming increasingly affordable; therefore, integrating VR into routine pediatric care could provide a scalable, non-pharmacological adjunct to existing anxiety-management protocols.

The present study examined the immediate impact of an immersive virtual reality (VR) experience on two objective indices of autonomic arousal in children—electrodermal activity (EDA) and blood volume pulse (BVP). Using a repeated-measures design with 120 paired observations, we found that VR markedly reduced physiological reactivity: the number of skin-conductance responses fell by 68% and the plethysmographic amplitude by 53%. Paired *t*-tests confirmed that both decreases were highly significant (*p* < 0.001) and were accompanied by very large effect sizes (d = 3.84 for EDA; d = 2.77 for BVP). These effect magnitudes far exceed the conventional benchmark for a “large” effect (d ≥ 0.80; Cohen) and indicate not merely statistical, but also clinical relevance [41]. These physiological findings translate to tangible benefits for pediatric patients undergoing dental procedures. The use of VR appears to create a more relaxed environment, potentially mitigating the fear and anxiety commonly associated with dental visits. By engaging children in immersive virtual experiences, VR effectively diverts their attention from the clinical setting, leading to reduced stress responses. This not only improves the immediate experience for the child but may also contribute to more positive long-term attitudes towards dental care. The consistent results across multiple VR sessions suggest that this intervention could be a valuable tool in pediatric dentistry, offering a non-pharmacological approach to anxiety management and potentially improving overall treatment outcomes.

The analysis also provides the following strong support for the study objectives:

1. Quantifying physiological effects: The data clearly shows a reduction in physiological stress indicators when VR is used. Electrodermal activity (EDA) measurements during VR sessions exhibit lower and more stable readings, with fewer peaks and lower amplitudes compared to the non-VR session. This indicates decreased sympathetic nervous system activation, suggesting improved comfort and relaxation. Blood volume pulse (BVP) data also demonstrate more consistent patterns in VR sessions, reflecting a calmer cardiovascular state. These physiological markers strongly suggest that VR improves patient comfort, relaxation, and potentially cooperation during dental procedures.
2. Comparing VR to traditional techniques: While the data does not directly compare VR to other traditional anxiety management techniques, the stark contrast between VR and non-VR sessions implies that VR could be a highly effective alternative or complementary method. The significant reduction in stress indicators during VR sessions suggests that this approach may be superior to some traditional methods in managing pediatric dental anxiety.
3. Supporting future applications: The consistent positive results across multiple VR sessions provide compelling preliminary evidence to support the broader adoption of VR in clinical pediatric dentistry. The clear physiological benefits observed in these simple dental procedures offer a strong foundation for future research. These data can guide investigations into VR’s long-term impacts on dental anxiety and its potential applicability in more invasive procedures. The quantifiable nature of the physiological improvements also provides a solid basis for developing standardized protocols for VR use in pediatric dentistry.

Exposure to an immersive VR environment produced a large, immediate reduction in autonomic arousal among children undergoing a potentially stressful clinical procedure. These findings provide compelling evidence that VR can serve as an effective and objective means of mitigating physiological stress responses and support the broader adoption of VR-based interventions in pediatric healthcare settings.

## Figures and Tables

**Table 1:** Descriptive statistics for electrodermal activity (EDA) peak counts and blood volume pulse (BVP) amplitudes at baseline (No-VR) and during the VR session.

		Mean	N	Std. Deviation	Std. Error Mean
Pair 1	EDA 1 No-VR	8.97	120	1.914	0.175
	EDA 2 VR	2.89	120	1.527	0.139
Pair 2	BVP 1 No-VR	300.00	120	100.201	9.147
	BVP 2 VR	140.38	120	60.238	5.499

**Table 2:** Paired-samples *t*-test results and effect sizes for EDA and BVP (N = 120).

		Paired Differences					t	df	Sig. (2-Tailed)	Cohen’s d
		Mean	Std. Deviation	Std. Error Mean	95% Confidence Interval of the Difference					
					Lower	Upper				
Pair 1	EDA 1–EDA 2	6.07	1.58	0.14	−3.84	6.36	42.03	119	0.00	−3.84
Pair 2	BVP 1–BVP 2	159.62	57.55	5.25	−2.77	170.02	30.38	119	0.00	−2.77

## Data Availability

The original contributions presented in this study are included in the article. Further inquiries can be directed to the corresponding authors.
